# Combining patient proteomics and in vitro cardiomyocyte phenotype testing to identify potential mediators of heart failure with preserved ejection fraction

**DOI:** 10.1186/s12967-016-0774-3

**Published:** 2016-01-20

**Authors:** Roseanne Raphael, Diana Purushotham, Courtney Gastonguay, Marla A. Chesnik, Wai-Meng Kwok, Hsiang-En Wu, Sanjiv J. Shah, Shama P. Mirza, Jennifer L. Strande

**Affiliations:** Cardiovascular Center, Medical College of Wisconsin, Milwaukee, WI USA; Department of Medicine, Medical College of Wisconsin, Milwaukee, WI USA; Division of Cardiovascular Medicine, Medical College of Wisconsin, Milwaukee, WI USA; MEB/CVC 4579, 8701 Watertown Plank Road, Milwaukee, WI 53226 USA; Biotechnology and Bioengineering, Medical College of Wisconsin, Milwaukee, WI USA; Department of Anesthesiology, Medical College of Wisconsin, Milwaukee, WI USA; Division of Cardiology, Department of Medicine, Northwestern University Feinberg School of Medicine, Chicago, IL USA

**Keywords:** Platelet proteome, Heart failure with preserved ejection fraction, Inflammation, S100A8, Induced pluripotent stem cell-derived cardiomyocytes

## Abstract

**Background:**

Heart failure with ejection fraction (HFpEF) is a syndrome resulting from several co-morbidities in which specific mediators are unknown. The platelet proteome responds to disease processes. We hypothesize that the platelet proteome will change composition in patients with HFpEF and may uncover mediators of the syndrome.

**Methods and results:**

Proteomic changes were assessed in platelets from hospitalized subjects with symptoms of HFpEF (n = 9), the same subjects several weeks later without symptoms (n = 7) and control subjects (n = 8). Mass spectrometry identified 6102 proteins with five scans with peptide probabilities of ≥0.85. Of the 6102 proteins, 165 were present only in symptomatic subjects, 78 were only found in outpatient subjects and 157 proteins were unique to the control group. The S100A8 protein was identified consistently in HFpEF samples when compared with controls. We validated the fining that plasma S100A8 levels are increased in subjects with HFpEF (654 ± 391) compared to controls (352 ± 204) in an external cohort (p = 0.002). Recombinant S100A8 had direct effects on the electrophysiological and calcium handling profile in human induced pluripotent stem cell-derived cardiomyocytes.

**Conclusions:**

Platelets may harbor proteins associated with HFpEF. S100A8 is present in the platelets of subjects with HFpEF and increased in the plasma of the same subjects. We further established a bedside-to-bench translational system that can be utilized as a secondary screen to ascertain whether the biomarkers may be an associated finding or causal to the disease process. S100A8 has been linked with other cardiovascular disease such as atherosclerosis and risk for myocardial infarction, stroke, or death. This is the first report on association of S100A8 with HFpEF.

**Electronic supplementary material:**

The online version of this article (doi:10.1186/s12967-016-0774-3) contains supplementary material, which is available to authorized users.

## Background

The platelet proteome is an untapped resource for identifying proteins that may reflect a disease process. Platelets are easily accessible and free from major highly abundant proteins making them an attractive model for proteomic studies. Platelets change the composition of their proteins in diseases such as Alzheimer’s, cancer, diabetes, coronary artery disease and acute coronary syndrome [1–4]. Platelets are largely under-studied in heart failure, yet evidence indicates that both platelet function [5, 6] and platelet-derived proteins such as adhesion molecules and the natriuretic peptide receptor-C [7–10] are altered in heart failure. Therefore, changes in the platelet proteome may allow for the identification of proteins that influence the disease process in heart failure.

Heart failure with preserved ejection fraction (HFpEF) affects almost 50 % of patients with heart failure and is increasing in prevalence [11], yet the pathophysiological mechanisms are poorly understood. HFpEF is associated with diabetes, hypertension, renal dysfunction, atrial fibrillation and obesity. The systemic inflammatory state induced by these co-morbidities is predictive of HFpEF [12, 13]. Platelets are both contributors and responders of inflammatory processes [14]. Considering there are no targeted therapies for HFpEF and morbidity and mortality are high, it is paramount to identify biomarkers associated with HFpEF and clarify their mechanistic role in clinical heart failure in order to develop targeted treatments. Consequently, by examining the platelet proteome of subjects with HFpEF, there is the potential to identify proteins that may provide insight into the disease mechanisms.

We established a novel bed-to-bench translational system to identify potential mediators of HFpEF using both platelet proteome analysis and mechanistic studies in induced pluripotent stem cell-derived cardiomyocytes. The broad utility of this strategy is to incorporate bioactivity studies into guiding the selection of proteins from proteomic studies for further investigation. We sought to compare the platelet proteome among subjects with HFpEF in the uncompensated (hospitalized) state, compensated (outpatient) state, and controls combined with validation in plasma samples from an external cohort and bioactivity studies using human induced pluripotent stem cell (iPSC)-derived cardiomyocytes. We hypothesized that [1] platelet proteomic analysis would successfully identify a protein associated with HFpEF, and [2] human iPSC-derived cardiomyocytes treated with recombinant proteins could serve as further validation by demonstrating phenotypic changes in cardiomyocyte calcium handling, which is altered in HFpEF.

## Methods

### Study population

For the discovery phase, subjects ≥50 years old presenting with New York Heart Association class II–III heart failure symptoms, a left ventricular ejection fraction (LVEF) >50 %, echocardiographic evidence of diastolic dysfunction and increased LV filling pressure were evaluated at the Medical College of Wisconsin between June 2012 to December 2013 for participation in this study. Increased LV filling pressures were defined as E/e′ ≥ 15, or E/e′ ≥ 8 and ≤ 15 with either a BNP ≥ 200 pg/ml or a left atrial (LA) volume index > 40 ml/m^2^. Subjects were excluded if they had a clinical condition that potentially changed the platelet or plasma proteomic profile independent of HFpEF such as uncontrolled diabetes, an active infection or inflammatory disorder, chronic renal failure requiring dialysis, severe liver disease, malignancy, acute myocardial infarction, chronic obstructive pulmonary disease requiring steroids, or recent surgical or invasive cardiac procedures. Subjects were excluded if they had other cardiac causes for their symptoms such as severe valvular disease, amyloidosis, or hypertrophic cardiomyopathy. Blood was drawn from the nine subjects enrolled in the study (HFpEF hospitalized group). Five of these subjects (HFpEF outpatient group) returned ≥2 weeks after discharge for second blood draw. Subjects with an LVEF ≥50 % and without evidence of increased LV filling pressures served as the control group.

For further biomarker validation, an additional set of 25 HFpEF subjects and 18 age and co-morbidity matched control subjects were recruited from Northwestern University. All subjects gave written informed consent to participate in the study. The Institutional Review Board at the Medical College of Wisconsin and Northwestern University approved the respective study protocols, which conformed to the principles of the Declaration of Helsinki.

### Reagents

Supplies and other reagents were purchased from Sigma-Aldrich (St. Louis, MO) unless specified. Recombinant S100A8 was purchased from Creative BioMart (Shirley, NY).

### Platelet preparation

Blood was separated into serum and platelet fractions. Platelets were extensively washed in buffer (45 mM sodium citrate, 25 mM citric acid, 80 mM d-glucose). During all steps, care was taken to avoid activation of platelets. Flow cytometry with anti-CD41 (Life Technologies, Grand Island, NY) and anti-P-selectin (BioLegend, San Diego, CA) was performed to assess for platelet activation (Additional file: 1. Figure S1). Microscopy confirmation verified that the purified platelets had leukocyte and red blood cell contamination that was less than 0.02 and 1 %, respectively (Additional file: 2. Figure S2).

### Global proteomic studies

Platelets from individual samples were resuspended in lysis buffer (125 mM Tris pH 6.8, 4 % SDS, 10 % glycerol, 5 % β-mercaptoethanol, Roche Complete Protease Inhibitor, Thermo HALT Phosphatase Inhibitor Cocktail). After determining protein concentration, the protein sample was separated by 1-dimensional SDS-PAGE gel (Bis-Tris 4–12 %) with internal DNA markers as described in our earlier publication [15]. The gel was stained with indoine blue and divided into three pieces. The proteins were reduced with 100 mM dithiotreitol (DTT) in 25 mM NH_4_HCO_3_ for 30 min at 56 °C and alkylated with 55 mM iodoacetamide (IAA) in 25 mM NH_4_HCO_3_ for 30 min at room temperature followed by trypsin digestion overnight. Peptides were extracted with 0.1 % trifluoroacetic acid (TFA) and 70 % acetonitrile/5 % TFA in water, respectively. Extracts were dried in a Speedvac and subsequently acidified to 0.1 % TFA. The samples were desalted using a ZipTip (C18).

For biomarker discovery, all samples were subject to tandem mass spectrometry. Three injection replicates of each fraction (three fractions per sample) were run on an LTQ-Orbitrap Velos mass spectrometer (Thermo Scientific). For each injection replicate, 1.5 µl sample was separated via C18 column over the course of a 150 min gradient from buffer A (2 % acetonitrile, 98 % H_2_O, 0.1 % formic acid) to buffer B (98 % acetonitrile, 2 % H_2_O, 0.1 % formic acid). The gradient program began with 2 min at 98 % A, followed by a 3 min ramp to 95 % A, a 115 min ramp to 60 % A, a 15 min ramp to 2 % A, 3 min at 2 % A, 2 min ramp to 98 % A, then a 10 min equilibration in 98 % A. MS1 scans were detected in the FTMS section of the Orbitrap Velos in profile mode at a resolution of 30,000 (full width of peak at half-maximum at 400 m/z). The ten most abundant parent ions from each MS1 scan were selected for fragmentation via collision induced dissociation. Results of SEQUEST searches against UniProt human database (version April 2013) and all nine runs of each sample were combined using Visualize software. Visualize software was also used to generate comparison data [16]. The protein lists include proteins identified with at least five scans that were observed with peptide probability >0.85.

### S100A8 expression

S100A8 levels were determined using a S100A8 enzyme-linked immunoassay kit from MBL International (Des Plaines, IL).

### Induced pluripotent stem cell induced-cardiomyocyte differentiation

The induced pluripotent stem cell (iPSC) line used in this study was a generous gift from Dr. Stephan Duncan. This iPSC line was generated from human foreskin fibroblasts and previously characterized [17]. The iPSC line was maintained on Matrigel (BD Biosciences, San Jose, CA) in mTeSR-1 media (Stem Cell Technologies, BC, Canada) and differentiated into cardiomyocytes according to published protocols [18, 19]. Differentiated cells were maintained in cardiomyocyte maintenance media (RPMI/B27; Life Technologies, Grand Island, NY). For all experiments, 35 ± 5 day old contracting cardiomyocytes were used.

### Electrophysiology

Action potentials were recorded from the human iPSC-derived cardiomyocytes using the current clamp configuration of the patch clamp technique, as previously described [20, 21]. Briefly, patch pipettes were pulled from borosilicate glass capillaries (King Precision Glass, Claremont, CA) with a micropipette puller (PC-10; Harishige, Tokyo, Japan) and heat polished using a microforge (MF-830; Narishige). The pipette resistances ranged from 3–5 MΩ when filled with the intracellular recording solution. This pipette solution contained 60 mM K-glutamate, 50 mM KCL, 10 mM HEPES, 1 mM MgCl_2_, 11 mM EGTA, 1 mM CaCl_2_, and 5 mM K_2_-ATP (pH adjusted to 7.4 with KOH). The extracellular bath solution contained 132 mM NaCl, 4.8 mM KCl, 1.2 mM MgCl_2_, 1.0 mM CaCl_2_, 5 mM dextrose, and 10 mM HEPES (pH adjusted 7.4 with NaOH). Action potentials were recorded using a Multiclamp 700B amplifier and Digidata 1440A interface (Molecular Devices, Sunnyvale, CA). pClamp 10 software (Molecular Devices) was used for data acquisition and analysis. Spontaneously beating nodal-, atrial-, and ventricular-like cells were characterized based on the maximum rate of depolarization (dV/dt), action potential duration (APD) at 50 and 90 % repolarization, and maximum diastolic potential. Recordings were conducted at physiological temperature (37 °C). The temperature of the recording chamber was controlled via a temperature control unit (TC 344B; Warner Instruments, Hamden, CT).

### Ratiometric Ca^2+^ microfluorometry

Briefly, human iPSC-derived cardiomyocytes plated on coverslips were exposed to Fura-2-AM (5 µM) for 30 min at room temperature, washed three times with extracellular bath solution, and given 30 min for de-esterification. For Ca^2+^ microfluorometry, the fluorophore was excited alternately with 340 and 380 nm wavelength illumination and images were acquired at 510 nm through a 20× objective. Recordings from each cell were obtained at a rate of 3 Hz. After background subtraction, the fluorescence ratio R for individual cell was determined as the intensity of emission during 340 nm excitation (I_340_) divided by I_380_, on a pixel-by-pixel basis. Activation-induced transients were generated by depolarization produced by microperfusion application of 50 mM KCl [22].

### Statistical analysis

Data is presented as either mean ± SD or as total percentage. Continuous variables were compared using the Student t test, assuming equal variance and dichotomous variables were compared using the Fisher exact test. Mass spectrometry measurements between groups were compared for either the presence (assigned a number value of 1) or absence (assigned a number of value of 0) of the protein identified in the sample using non-parametric Wilcoxon rank-sum tests without adjusting for multiple testing. Mass spectrometry data analysis was performed by the biostatical consulting service at the Medical College of Wisconsin.

## Results

### Clinical and echocardiographic characteristics of the discovery cohort

As described in Table 1 the median age of the HFpEF subjects is slightly greater than the control subjects (p = 0.04). The HFpEF group had a higher incidence of atrial fibrillation and cerebral vascular accident/transient ischemia in comparison to control subjects. Although not statistically significant, HFpEF subjects were more likely to have diabetes, coronary heart disease, hyperlipidemia and a distant smoking history. A significant number of HFpEF subjects were taking beta blockers compared to the control group. Echocardiogram studies confirmed the presence of diastolic dysfunction and increased LV pressure in the HFpEF group (Table 2). Left atrial volume indices were significantly elevated along with an increase in LV wall thickness in the HFpEF group compared to control.

**Table 1:** Clinical characteristics of subjects

Characteristic	HFpEF (n = 9)	Control (n = 7)	p value <0.05
Age, years	75 ± 10	62 ± 13	0.03
Women (%)	75	71	n.s.
Body mass index	33 ± 9	33 ± 10	n.s.
Hypertensive (%)	67	75	n.s.
Hyperlipidemia (%)	67	63	n.s.
Diabetes (%)	56	25	n.s.
Coronary artery disease (%)	56	29	n.s.
h/o CVA/TIA (%)	50	0	0.02
h/o Afib (%)	78	0	<0.001
Smoking history (%)	100	29	<0.001
Current smoker (%)	11	14	n.s.
Former smoker (%)	89	14	n.s.
Medications			
ACEI/ARB (%)	50	57	n.s
Beta-blocker (%)	100	50	0.009
Aldosterone antagonist (%)	0	0	n.s.
Statin (%)	75	57	n.s.
Diuretic (%)	44	43	n.s.

*h/o* history of; *CVA/TIA* cerebral vascular accident/transient ischemic attack, *Afib* atrial fibrillation, *ACEI/ARB* angiotensin converting enzyme inhibitor/angiotensin receptor blocker

The p value was calculated using two tailed student t-tests for numerical variables and using Chi squared and Fisher’s exact tests for categorical values

**Table 2:** Echocardiographic characteristics of subjects

Characteristic	HFpEF (n = 9)	Control (n = 7)	p value <0.05
2D Echocardiography			
LA volume index, ml/m^2^	49 ± 15	32 ± 7.7	0.018
LV internal diameter, cm	4.64 ± 0.37	4.73 ± 0.08	NS
Interventricular septum, cm	1.25 ± 0.12	0.92 ± 0.01	0.001
Posterior wall, cm	1.20 ± 0.18	0.88 ± 0.09	0.004
LV mass index, g/m^2^	112 ± 20	90 ± 45	NS
Ejection fraction, %	55 ± 6	60 ± 3	NS
Doppler data			
E peak, cm/s	86.6 ± 26	68.0 ± 5.8	NS
e′ peak	6.9 ± 1.66	7.7 ± 1.03	NS
E/e′ ratio	14.4 ± 5.13	9.28 ± 0.37	NS
Diastolic dysfunction, %	100	14	<0.001

*LA* left atrium, *LV* left ventricle

The p value was calculated using two-tailed student t-tests

### Overall description of proteomic findings

**Fig. 1:**
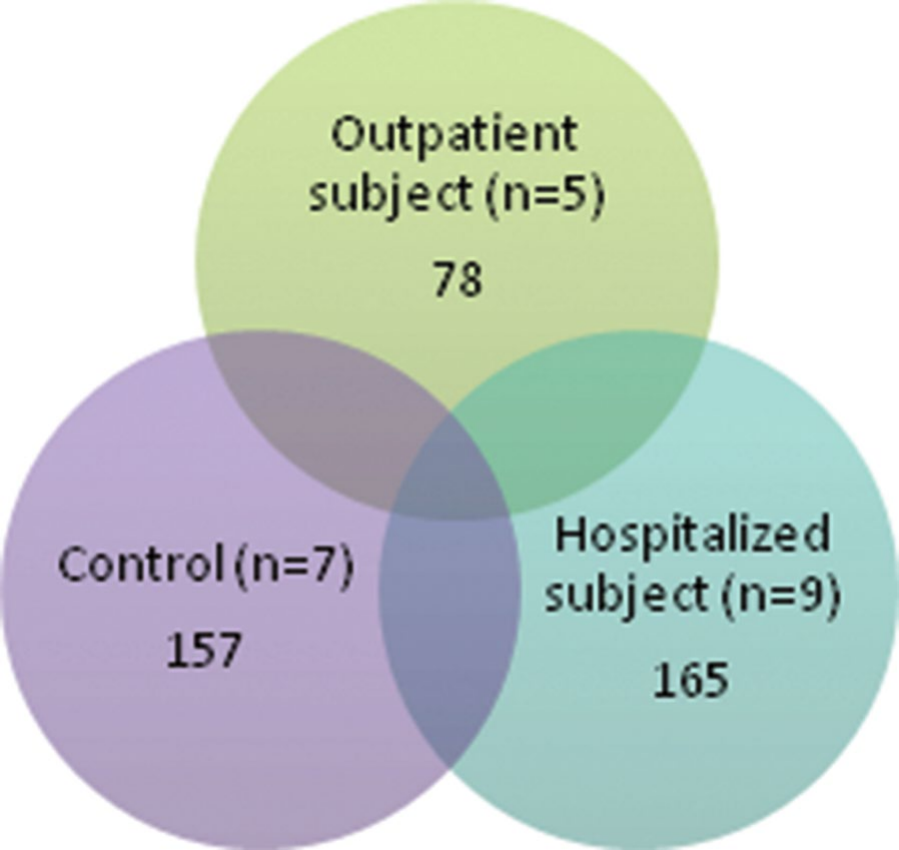
Global proteomic analysis of platelets identifies 6102 proteins. The Venn diagram displays he results of the analysis of platelet proteins from the individual subjects by in-depth LC–MS/MS. In total, 6102 proteins were identified with 4172 common among all data sets. There were 165, 78, and 157 proteins identified that were unique to the HFpEF Symptomatic, HFpEF outpatient and Control groups

Global proteomic experiments were performed using 21 separate platelet preparations. Combining these experiments, a total of 6102 proteins were identified with at least five scans with a protein probability of >0.85. The HFpEF hospitalized group had a total of 5546 proteins, the HFpEF outpatient group had a total of 4854 proteins and the control group had a total of 5498 proteins identified. A total of 4172 proteins were found to be shared among all three groups. When comparing two groups, 321 proteins were identified as being shared amongst the outpatient and control group. A total of 361 proteins were found in both the hospitalized and outpatient groups and a total of 848 proteins were found in both the control and hospitalized groups. The number of unique proteins in each group consisted of 165 proteins in the HFpEF hospitalized group, 78 proteins in the HFpEF outpatient group, and 157 unique proteins in the control group (Fig. 1). To assess for possible contamination from other blood cells, the data set was scanned for the presence of CD45 and MHC II chains; proteins that are expressed in leukocytes. These proteins were not found in the data set; therefore, the contamination from leukocytes was likely to be minimal. However, complement C5 and β-2-glycoprotein were identified in the data sets denoting some serum contamination was present.

### Unique proteins in each study group

The platelet proteome from nine subjects were analyzed in the HFpEF hospitalized group, five subjects in the HFpEF outpatient and seven subjects in the control group. The unique proteins identified with a scan count of >9 are listed in Table 3. In addition after applying the non-parametric Wilcoxon rank-sum test, 37 proteins were found to be more prevalent amongst the combined HFpEF groups than with the control and 77 proteins were identified that were found to be more prevalent amongst the control with a p value <0.05. These proteins are listed Table 4.

**Table 3:** List of unique proteins identified in each group with >9 scans total

Protein	Accession	Description
Present only in HFpEF symptomatic group		
NALP2	Q9NX02	NACHT, LRR and PYD domains-containing protein
ZEP3	Q5T1R4	Transcription factor HIVEP3
MET25	Q8N6Q8	Methyltransferase-like protein 25
SCAF8	Q9UPN6	Protein SCAF8
CC105	Q8IYK2	Coiled-coil domain-containing protein 105
FILA	P20930	Filaggrin
MEG11	A6BM72	Multiple epidermal growth factor-like domains protein 11
F19A2	Q8N3H0	Protein FAM19A2
GRM1	Q13255	Metabotropic glutamate receptor
YP010	Q96M66	Putative uncharacterized protein FLJ32790
PSMD4	P55036	26S proteasome non-ATPase regulatory subunit 4
PCCA	P05165	Propionyl-CoA carboxylase alpha chain, mitochondrial
TCPR2	O15040	Tectonin beta-propeller repeat-containing protein
KPRP	Q5T749	Keratinocyte proline-rich protein
GTPB5	Q9H4K7	GTP-binding protein 5
CV031	O95567	Uncharacterized protein C22orf31
TFB2 M	Q9H5Q4	Dimethyladenosine transferase 2, mitochondrial
SPXN4	Q5MJ08	Sperm protein associated with the nucleus on the X chromosome N4
PF21A	Q96BD5	PHD finger protein 21A
Present only in HFpEF asymptomatic group		
H2A1H	Q96KK5	Histone H2A type
H2A3	Q7L7L0	Histone H2A type 3
POK7	Q9QC07	HERV-K_1q23.3 provirus ancestral Pol protein
CC127	Q96BQ5	Coiled-coil domain-containing protein 127
CC85C	A6NKD9	Coiled-coil domain-containing protein 85C
WDR75	Q8IWA0	WD repeat-containing protein 75
CXCL3	P19876	C-X-C motif chemokine 3
RGPS1	Q5JS13	Ras-specific guanine nucleotide-releasing factor RalGPS1
CXCL2	P19875	C-X-C motif chemokine 2
CHMP7	Q8WUX9	Charged multivesicular body protein 7
CK2N2	Q96S95	Calcium/calmodulin-dependent protein kinase II inhibitor 2
CHIT1	Q13231	Chitotriosidase-1
NOX1	Q9Y5S8	NADPH oxidase 1
RBY1C	P0DJD4	RNA-binding motif protein, Y chromosome, family 1 member C
WFDC3	Q8IUB2	WAP four-disulfide core domain protein 3
ABCBB	O95342	Bile salt export pump
HHAT	Q5VTY9	Protein-cysteine N-palmitoyltransferase HHAT
MID51	Q9NQG6	Mitochondrial dynamic protein MID51
LMNB1	P20700	Lamin-B1
Present only in control group		
MY15B	Q96JP2	Putative unconventional myosin-XVB
CC020	Q8ND61	Uncharacterized protein C3orf20
MCTS1	Q9ULC4	Malignant T cell-amplified sequence 1
KSR1	Q8IVT5	Kinase suppressor of Ras 1
PRP6	O94906	Pre-mRNA-processing factor 6
DDX59	Q5T1V6	Probable ATP-dependent RNA helicase DDX59
AL1A3	P47895	Aldehyde dehydrogenase family 1 member A3
PCCB	P05166	Propionyl-CoA carboxylase beta chain, mitochondrial
HNRCL	O60812	Heterogeneous nuclear ribonucleoprotein C-like 1
BIRC3	Q13489	Baculoviral IAP repeat-containing protein 3
NDUF4	Q9P032	NADH dehydrogenase 1 alpha subcomplex assembly factor 4
MIRO2	Q8IXI1	Mitochondrial Rho GTPase 2
Present in HFpEF symptomatic and HFpEF asymptomatic groups but not control group		
MBD5	Q9P267	Methyl-CpG-binding domain protein 5
RRBP1	Q9P2E9	Ribosome-binding protein 1
ZNF79	Q15937	Zinc finger protein 79
DCNL5	Q9BTE7	DCN1-like protein 5
RGS3	P49796	Regulator of G-protein signaling 3
TMOD2	Q9NZR1	Tropomodulin-2
MYO5B	Q9ULV0	Unconventional myosin-Vb
SC24D	O94855	Protein transport protein Sec24D
SHIP1	Q92835	Phosphatidylinositol 3,4,5-trisphosphate 5-phosphatase 1
ASIC1	P78348	Acid-sensing ion channel 1
DMXL1	Q9Y485	DmX-like protein 1
RECQ1	P46063	ATP-dependent DNA helicase Q1
LY10L	Q9H930	Nuclear body protein SP140-like protein
MBNL1	Q9NR56	Muscleblind-like protein 1
KCC2B	Q13554	Calcium/calmodulin-dependent protein kinase type II subunit beta
LIPA3	O75145	Liprin-alpha-3
CD109	Q6YHK3	CD109 antigen
ZN141	Q15928	Zinc finger protein 141
YTHD2	Q9Y5A9	YTH domain family protein 2
PLCD	Q9NRZ5	1-acyl-sn-glycerol-3-phosphate acyltransferase delta
KIFA3	Q92845	Kinesin-associated protein 3
TRI25	Q14258	E3 ubiquitin/ISG15 ligase TRIM25
ETUD1	Q7Z2Z2	Elongation factor Tu GTP-binding domain-containing protein 1
CDN1B	P46527	Cyclin-dependent kinase inhibitor 1B
CO4A4	P53420	Collagen alpha-4(IV) chain
TEX35	Q5T0J7	Testis-expressed sequence 35 protein
MUC16	Q8WXI7	Mucin-16
NPIL2	A6NJ64	NPIP-like protein LOC729978
IRF2	P14316	Interferon regulatory factor 2
MK07	Q13164	Mitogen-activated protein kinase 7
APOA	P08519	Apolipoprotein(a)
HIBCH	Q6NVY1	3-hydroxyisobutyryl-CoA hydrolase, mitochondrial
USH1C	Q9Y6N9	Harmonin
GOG8O	A6NCC3	Golgin subfamily A member 8O
NADE	Q6IA69	Glutamine-dependent NAD(+) synthetase
MET17	Q9H7H0	Methyltransferase-like protein 17, mitochondrial
PITH1	Q9GZP4	PITH domain-containing protein 1
IL1R1	P14778	Interleukin-1 receptor type 1
C1GLT	Q9NS00	Glycoprotein-N-acetylgalactosamine 3-beta-galactosyltransferase 1
OR2L3	Q8NG85	Olfactory receptor 2L3
KV122	P04430	Ig kappa chain V-I region BAN
GG8L2	A6NP81	Golgin subfamily A member 8-like protein 2
ZFYV1	Q9HBF4	Zinc finger FYVE domain-containing protein 1
CJ076	Q5T2E6	UPF0668 protein C10orf76
STAB 1	Q9NY15	Stabilin-1
EHBP1	Q8NDI1	EH domain-binding protein 1
ANR24	Q8TF21	Ankyrin repeat domain-containing protein 24
FAHD1	Q6P587	Acylpyruvase FAHD1, mitochondrial
IWS1	Q96ST2	Protein IWS1 homolog
THAP2	Q9H0W7	THAP domain-containing protein 2
FNIP1	Q8TF40	Folliculin-interacting protein 1
STK16	O75716	Serine/threonine-protein kinase 16
CXX1	O15255	CAAX box protein 1
GOG8R	I6L899	Golgin subfamily A member 8R
SRRT	Q9BXP5	Serrate RNA effector molecule homolog
ZN611	Q8N823	Zinc finger protein 611
MRE11	P49959	Double-strand break repair protein MRE11A
LONM	P36776	Lon protease homolog, mitochondrial
GOG8 N	F8WBI6	Golgin subfamily A member 8 N
ALPK2	Q86TB3	Alpha-protein kinase 2
EI2BG	Q9NR50	Translation initiation factor eIF-2B subunit gamma
NBPFL	A6NDD8	Neuroblastoma breakpoint family member 21
ETV7	Q9Y603	Transcription factor ETV7

**Table 4:** Proteins preferential to either HFpEF or control groups

Protein	Accession	Description	p value
Proteins preferentially found in HFpEF group			
SAA2	P0DJI9	Serum amyloid A-2 protein	0.0019
SAA1	P0DJI8	Serum amyloid A-1 protein	0.0019
PHF3	Q92576	PHD finger protein 3	0.0090
RGPD5	Q99666	RANBP2-like and GRIP domain-containing protein 5/6	0.0123
RGPD8	O14715	RANBP2-like and GRIP domain-containing protein 8	0.0124
YMEL1	Q96TA2	ATP-dependent zinc metalloprotease YME1L1	0.0256
FHR2	P36980	Complement factor H-related protein 2	0.0269
RGPD3	A6NKT7	RanBP2-like and GRIP domain-containing protein 3	0.0278
CG010	Q9HAC7	CaiB/baiF CoA-transferase family protein C7orf10	0.0279
RRBP1	Q9P2E9	Ribosome-binding protein 1	0.0279
ZNF79	Q15937	Zinc finger protein 79	0.0279
DCNL5	Q9BTE7	DCN1-like protein 5	0.0279
RECQ1	P46063	ATP-dependent DNA helicase Q1	0.0283
PERQ2	Q6Y7W6	PERQ amino acid-rich with GYF domain-containing protein 2	0.0285
MBD5	Q9P267	Methyl-CpG-binding domain protein 5	0.0286
GPCP1	Q9NPB8	Glycerophosphocholine phosphodiesterase GPCPD1	0.0286
NOL10	Q9BSC4	Nucleolar protein 10	0.0351
LBP	P18428	Lipopolysaccharide-binding protein	0.0432
AFF1	P51825	AF4/FMR2 family member 1	0.0442
SOX30	O94993	Transcription factor SOX-30	0.0458
DCP1A	Q9NPI6	mRNA-decapping enzyme 1A	0.0465
AN20B	Q5CZ79	Ankyrin repeat domain-containing protein 20B	0.0468
TCOF	Q13428	Treacle protein	0.0479
MEN1	O00255	Menin	0.0486
S10A8	P05109	S100A8	0.0808
Proteins preferentially found in control group			
MY15B	Q96JP2	Putative unconventional myosin-XVB	0.0012
ASXL3	Q9C0F0	Putative Polycomb group protein ASXL3	0.0045
CC020	Q9NX02	NACHT, LRR and PYD domains-containing protein 2	0.0045
TEKT1	Q969V4	Tektin-1	0.0070
SEP10	Q9P0V9	Septin-10 OS = Homo sapiens	0.0103
LMNB2	Q03252	Lamin-B2 OS = Homo sapiens	0.0103
ZN469	Q96JG9	Zinc finger protein 469	0.0146
PARI	Q9NWS1	PCNA-interacting partner	0.0148
NOP2	P46087	Putative ribosomal RNA methyltransferase NOP2	0.0148
FIGL2	A6NMB9	Putative fidgetin-like protein 2	0.0148
MCTS1	Q9ULC4	Malignant T-cell-amplified sequence 1	0.0148
TANC2	Q9HCD6	Protein TANC2	0.0148
HEM0	P22557	5-aminolevulinate synthase, erythroid-specific, mitochondrial	0.0148
PRP6	O94906	Pre-mRNA-processing factor 6	0.0148
TACC2	O95359	Transforming acidic coiled-coil-containing protein 2	0.0200
SMC3	Q9UQE7	Structural maintenance of chromosomes protein 3	0.0261
GTF2I	P78347	General transcription factor II-I	0.0262
CI084	Q5VXU9	Uncharacterized protein	0.0268
CCS	O14618	Copper chaperone for superoxide dismutase	0.0294
COX6C	P09669	Cytochrome c oxidase subunit 6C	0.0324
INT11	Q5TA45	Integrator complex subunit 11	0.0352
DCLK1	O15075	Serine/threonine-protein kinase DCLK1	0.0363
SSH1	Q8WYL5	Protein phosphatase Slingshot homolog 1	0.0380
PJA1	Q8NG27	E3 ubiquitin-protein ligase Praja-1	0.0390
BRK1	Q8WUW1	Protein BRICK1	0.0422
UBP44	Q9H0E7	Ubiquitin carboxyl-terminal hydrolase 44	0.0422
PLCG2	P16885	1-phosphatidylinositol 4,5-bisphosphate phosphodiesterase gamma-2	0.0428
IGS22	Q8N9C0	Immunoglobulin superfamily member 22	0.0431
RPGFL	Q9UHV5	Rap guanine nucleotide exchange factor-like 1	0.0431
CN070	Q86TU6	Putative uncharacterized protein encoded by LINC00523	0.0431
TRI35	Q9UPQ4	Tripartite motif-containing protein 35	0.0431
TOPB1	Q92547	DNA topoisomerase 2-binding protein 1	0.0431
R3HD4	Q96D70	R3H domain-containing protein 4	0.0431
ABR	Q12979	Active breakpoint cluster region-related protein	0.0431
ZN441	Q8N8Z8	Zinc finger protein 441	0.0431
ZN451	Q9Y4E5	Zinc finger protein 451	0.0431
DCE2	Q05329	Glutamate decarboxylase 2	0.0431
RAB31	Q13636	Ras-related protein Rab-31	0.0431
PDE3A	Q14432	cGMP-inhibited 3′, 5′-cyclic phosphodiesterase A	0.0431
TRPM2	O94759	Transient receptor potential channel subfamily M member 2	0.0431
C163B	Q9NR16	Scavenger receptor cysteine-rich type 1 protein M160	0.0431
CA094	Q6P1W5	Uncharacterized protein C1orf94	0.0431
RSBN1	Q5VWQ0	Round spermatid basic protein 1	0.0431
GRM8	O00222	Metabotropic glutamate receptor 8	0.0431
KLHL7	Q8IXQ5	Kelch-like protein 7	0.0431
SHAN3	Q9BYB0	SH3 and multiple ankyrin repeat domains protein 3	0.0431
TTI1	O43156	TELO2-interacting protein 1 homolog	0.0431
FMO4	P31512	Dimethylaniline monooxygenase [N-oxide-forming] 4	0.0431
RARB	P10826	Retinoic acid receptor beta	0.0431
UTY	O14607	Histone demethylase UTY	0.0431
SLK	Q9H2G2	STE20-like serine/threonine-protein kinase	0.0431
RB39B	Q96DA2	Ras-related protein Rab-39B	0.0435
RB43L	A6NDJ8	Putative Rab-43-like protein	0.0435
RAB4B	P61018	Ras-related protein Rab-4B	0.0435
RAB12	Q6IQ22	Ras-related protein Rab-12	0.0435
RAB43	Q86YS6	Ras-related protein Rab-43	0.0435
RAB30	Q15771	Ras-related protein Rab-30	0.0435
GRM7	Q14831	Metabotropic glutamate receptor 7	0.0435
ZNF67	Q15940	Putative zinc finger protein 726P1	0.0435
FAKD5	Q7L8L6	FAST kinase domain-containing protein 5	0.0435
ZNF98	A6NK75	Zinc finger protein 98	0.0435
MFSD9	Q8NBP5	Major facilitator superfamily domain-containing protein 9	0.0435
RECK	O95980	Reversion-inducing cysteine-rich protein with Kazal motifs	0.0435
AL1A3	P47895	Aldehyde dehydrogenase family 1 member A3	0.0435
VP37C	A5D8V6	Vacuolar protein sorting-associated protein 37C	0.0435
ZN492	Q9P255	Zinc finger protein 492	0.0435
VPS29	Q9UBQ0	Vacuolar protein sorting-associated protein 29	0.0435
HNRCL	O60812	Heterogeneous nuclear ribonucleoprotein C-like 1	0.0435
DHRS7	Q9Y394	Dehydrogenase/reductase SDR family member 7	0.0452
BRD8	Q9H0E9	Bromodomain-containing protein 8	0.0455
IF2P	O60841	Eukaryotic translation initiation factor 5B	0.0455
GDPD3	Q7L5L3	Glycerophosphodiesterase domain-containing protein 3	0.0456
SYSC	P49591	Serine–tRNA ligase, cytoplasmic	0.0465
NEK9	Q8TD19	Serine/threonine-protein kinase Nek9	0.0473

p values are calculated based on the non-parametric Wilcoxon rank-sum tests

### Discovery and validation cohort ELISA confirmation

**Fig. 2:**
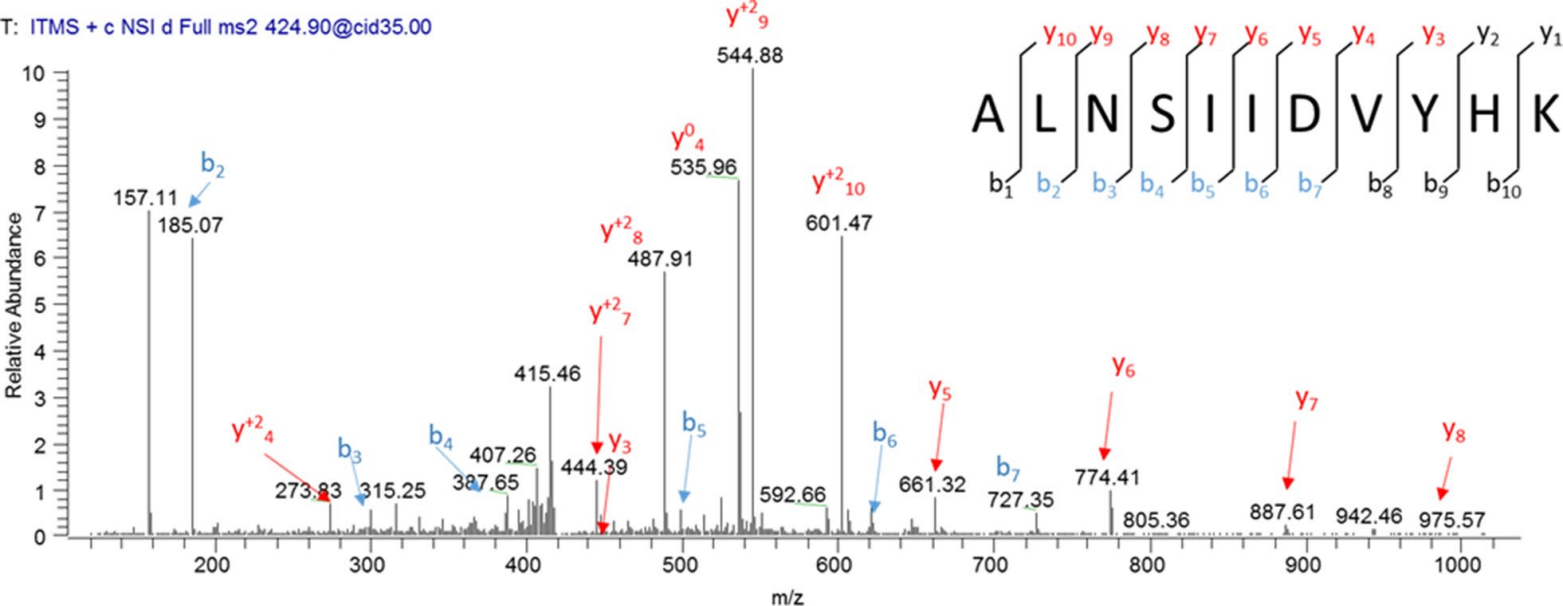
Representative MS/MS scan for S100A8 peptide sequence ALNSIIDVYHK. Raw m/z spectral images with peak assignments and *b* and *y* *ion* lists along with a representation of peptide sequencing by tandem mass spectrometry

**Fig. 3:**
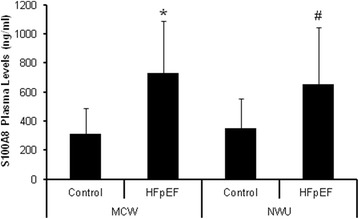
Plasma levels of S100A8 in control vs. HFpEF groups. a S100A8 is found in increased levels in the plasma of subjects with HFpEF vs. control subjects as detected by ELISA. The MCW columns include the control (n = 7) and HFpEF (n = 9) from the discovery cohort and the NWU colums include the control (n = 18) and HFpEF (n = 25) samples from the validation cohort. *p < 0.006 vs MCW Control.^ #^ *p* < 0. 002 vs NWU Control

One particularly interesting finding was the identification of S100A8. The m/z ratio graph representing S100A8 is shown in Fig. 2. Even though the p value was 0.08, it was identified in six out of the nine HFpEF subjects. S100A8 has not been previously associated with HFpEF but has been linked to advanced heart failure [23]. Additionally, S100A8 has been found to correlate with traditional cardiovascular risk factors and the manifestation of cardiovascular disease [24, 25]. For these reasons, we decided to look more closely at S100A8 to verify its association with HFpEF. S100A8 is found in platelets [26, 27] and the plasma [25, 28]; because we used the platelet lysates for the mass spect analysis, we used the plasma samples for quantitative ELISA analysis. Figure 3 shows that plasma S100A8 levels are increased symptomatic HFpEF when compared to control (MCW cohort). We then validated these findings by studying a larger cohort of subjects recruited from the Northwestern University HFpEF Program. In this larger cohort, we saw a similar increase in plasma S100A8 levels in the HFpEF group (Fig. 3; Northwestern cohort).

### Exogenously applied rS100A8 affects cardiomyocyte function in vitro

**Fig. 4:**
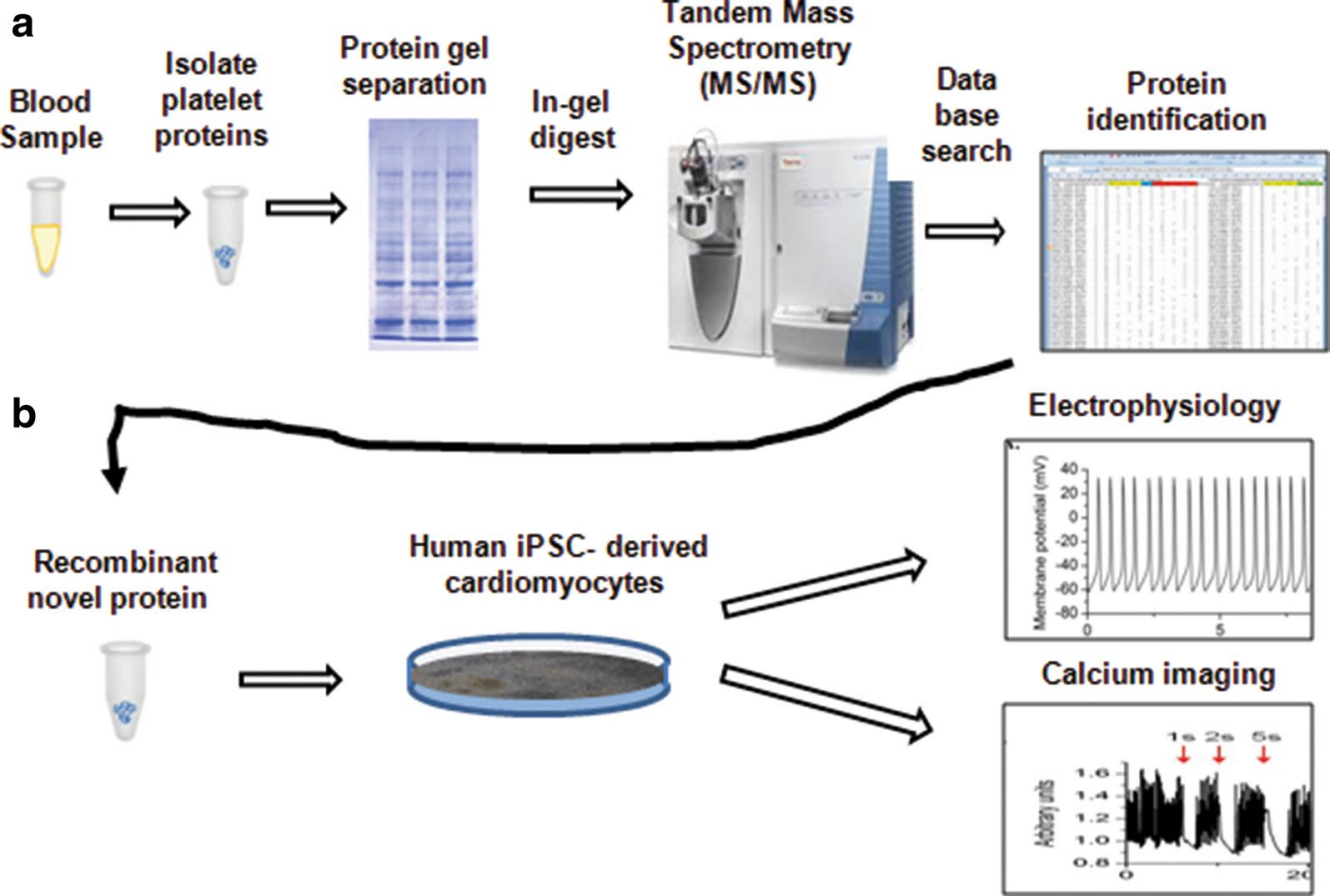
Overview of primary and secondary screening methods to identify potential mediators of HFpEF. **a** Platelet proteomes were subject to mass spectral analysis and novel proteins were identified. **b** Human cardiomyocytes derived from induced pluripotent stem cells were used to determine whether proteins that were identified in **a** had direct effects on cardiomyocytes function in vitro. Purified recombinant protein S100A8 was tested in this assay

To ascertain whether S100A8 may play a causal role in the HFpEF disease process; we developed a bedside-to-bench translational system (Fig. 4) to screen for biological effects of identified proteins on cardiomyocyte function in vitro. We added recombinant S100A8 (800 ng/ml) to iPSC-derived cardiomyocytes in vitro and measured action potentials and intracellular Ca^2+^ concentrations separately. This specific concentration of rS100A8 was selected as it was the average plasma concentration observed in the HFpEF group (Fig. 3).

**Fig. 5:**
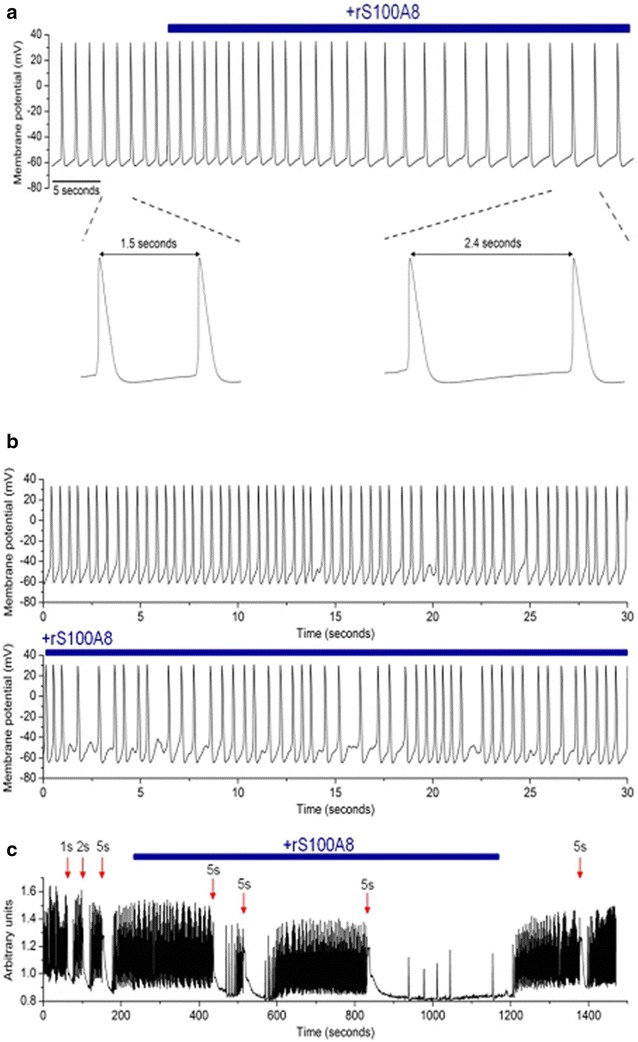
S100A8-mediated effects on human iPSC-derived cardiomyocytes. **a** Shows example action potentials recorded from rS100A8 treated iPSC derived human cardiomyocytes. The addition of rS100A8 to the buffer extended the period between action potentials. This period is phase 4; the diastolic membrane potential between action potentials. **b** rS100A8 exacerbates the arrhythmic tendencies of human cardiomyocytes. **c** Spontaneous Ca^2+^ transients recorded from human cardiomyocytes treated with rS100A8 as indicated by the blue line. rS100A8 significantly delayed the recovery of depolarization. Wash out of rS100A8 reversed these effects

Action potentials (APs) were recorded in the current clamp mode using the patch clamp technique. The recordings were acquired from spontaneously beating cells. External application of rS100A8 slowed the spontaneous pacing within 25 s which suggests the interaction with a membrane receptor. In the example shown in Fig. 5a, the spontaneous generation of APs with atrial-like properties was slowed in the presence of rS100A8. The peak-to-peak AP interval increased from 1.5 to 2.4 s. This effect was reversible upon washout of rS100A8 (results not shown). In a different beating cell cluster, the recorded atrial-like APs showed arrhythmogenic tendencies characterized by infrequent incidents of failed triggering of APs, as shown in Fig. 5b. The rS100A8 exacerbated this trend by increasing the frequency of these failed events. Thus, the electrophysiological profile of these iPSC-derived cardiomyocytes is profoundly impacted by rS100A8.

Intracellular Ca^2+^ concentrations ([Ca^2+^]_i_) were measured using the ratiometric Ca^2+^ microfluorometry technique with Fura-2-AM fluorescent dye. The [Ca^2+^]_i_ were monitored in spontaneously beating cells. The sample trace (Fig. 5c) shows a spontaneous Ca^2+^ transient recording that was interrupted by activity-induced depolarization (50 mM K^+^; duration of application as noted) at certain time points (indicated by the red arrows) using a microperfusion system. Of particular note is the recovery of the spontaneous Ca^2+^ transient following each depolarizing pulse. In the absence of rS100A8, the recovery was relatively fast. In contrast, the recovery was considerably slower in the presence of rS100A8. Following a third depolarizing pulse, recovery was not evident until the washout of rS100A8; this observation also suggests that rS100A8 effects are mediated through a membrane receptor. In summary, rS100A8 adversely affected the calcium handling of iPSC-derived cardiomyocytes.

## Conclusions

The key finding of this study was that it was possible to derive platelet protein data sets specific for HFpEF patients. These proof-of-concept findings suggest that the platelet proteome might provide a useful tool for screening for HFpEF-associated biomarkers. Although several platelet proteins were identified in HFpEF subjects; their exact connection to HFpEF has yet to be determined. Though our data is limited by the small size, our discovery cohort has similar characteristics of larger HFpEF cohorts reported in the literature [29–31]. By combining proteomics with bioactivity assays, we have demonstrated that the platelet proteome is an untapped resource for determining disease mediators in HFpEF.

The platelet proteome in healthy individuals is remarkably stable with only minor differences in protein expression patterns [32]. Veitinger et al. suggests the difference in platelet proteins between individuals is a results of the uptake of plasma proteins by the platelet [33]. Inflammation is closely linked with HFpEF [34] and considering that platelets are involved in the inflammatory process, it is not surprising that our proteomics screen led to the identification of several proteins also involved in inflammation. These include serum amyloid A (SAA), Lipopolysaccharide binding protein, apolipoprotein A1 and S100A8. Two proteins, serum amyloid-A (SAA) protein 1 and apolipoprotein A1 were increased in the sera of non-human primates after drug-induced cardiac injury [35]. In addition, increased levels of SAA in serum have been associated with coronary heart disease [36], as well as systolic heart failure [37] and has been shown to be a predictor of cardiovascular outcomes in women [38].

S100A8 is a member of the S100 calcium-binding family of proteins, which exhibit increased levels in a number of inflammatory states. S100A8 is commonly mentioned with its binding partner, S100A9. Even though S100A8 is found in the plasma [23], it is known that platelets and megakaryocytes might serve as an additional source of S100A8 and might contribute to the plasma pool of S100A8/A9 in inflammatory diseases and cardiovascular events [26, 27, 39].

S100A8 and S100A9 are not normally expressed in cardiomyocytes [40] although its cardiac expression can be induced by endotoxins or angiotensin II [40, 41]. Release of S100A8/A9 from cells allows it to act in a paracrine or autocrine fashion. These extracellular functions are mediated by the toll-like receptor 4 (TLR4) [42, 43] or the receptor for advanced glycation end products (RAGE) [40, 44, 45]. More recently, CD36 has been identified as a receptor [26]. In the mouse, S100A8/A9 signals through RAGE to promote inflammation and fibrosis after angiotensin II or hypoxic-induced cardiac injury [41, 45].

Increased platelet S100A8 mRNA and plasma protein levels were present in patients with acute myocardial infarction [39]. Plasma levels of S100A8/A9 predicted risk of future myocardial infarction, stroke or death in post-menopausal healthy women [25]. Elevated S100A8 levels have also been found in other inflammatory disorders which are associated with abnormalities of vascular and cardiac function, particularly diastolic dysfunction, such as diabetes [46–48], end-stage renal disease [49, 50], and inflammatory bowel disease [51, 52]. This is the first association of S100A8 with HFpEF, yet its role in the disease process still needs be elucidated. S100A8 has immediate effects on the electrophysiological and Ca^2+^ handling profiles of human induced cardiomyocytes suggesting that S100A8 is acting through a membrane receptor. S100A8 interaction with RAGE affects calcium flux in neonatal rat ventricular cardiomyocytes and HL-1 cardiomyocytes [40, 53]. The adverse effects on the electrophysiological and Ca^2+^ handling profiles resulting from S100A8 treatment of human induced cardiomyocytes; validates our bedside-to-bench translational screen as an approach to identify bioactive proteins that may contribute to the disease mechanisms in HFpEF.

We also considered the possibility that subjects progress to HFpEF through loss of cardioprotective proteins. Therefore, we searched amongst our control group and were able to identify four proteins that could potentially have protective qualities against the development of heart failure. Cyclic nucleotide phosphodiesterase 3A1 (PDE3A) regulates β-adrenergic signaling to effect physiological cardiac performance. Furthermore, PDE3A protects the heart against angiotensin II-induced cardiac remodeling in mice [54]. Copper Chaperone for Superoxide Dismutase (CCS) plays a role in copper delivery to tissues; disturbances in copper homeostasis mediates cardiomyopathy [55]. Zinc finger protein 451 a negative regulator of TGF-beta signaling [56]. The transient receptor potential cation channel subfamily M member 2 (TRPM2) protein limits oxidative stress injury and dampens the inflammatory response [57].

The present study must be interpreted within the context of its limitations. First of all, this was a discovery effort and not designed as a quantitative proteomic analysis. Therefore, we cannot determine if specific proteins are up- or down-regulated. In addition, it is unlikely that one protein is responsible for a complex disease as HFpEF, but our findings offer new perspectives regarding HFpEF and further confirmation of the platelet proteins identified in this study will need to be validated in a larger cohort. In addition, combining proteomics with functional bioactivity assessments may be a strategy to complement and strengthen the search for biomarkers by combining protein identified with biological activity in a relevant in vitro model system.

In conclusion, from the discovery set in HFpEF patients, we derived a panel of platelet proteins that may be specific for HFpEF. Furthermore, this set distinguished a set of platelet proteins which are consistent in HFpEF subjects whether they are decompensated and hospitalized or compensated after discharge. We further established a bedside-to-bench translational system that can be utilized as a secondary screen to ascertain whether the biomarkers may be an associated finding or causal to the disease process.

## Additional files

10.1186/s12967-016-0774-3 Flow cytometry to assess platelet activation. Purified platelet samples were incubated with fluorescently labeled antibodies against CD62P and CD41 and subject to flow cytometry. Platelets positive for CD41 + but negative for CD62P are non-activated. CD62P positive platelets are activated.
10.1186/s12967-016-0774-3 Microscopy for Platelet Purity. Isolated platelets were observed under the microscope. Visible red blood cells and leukocytes were counted and calculated as a percentage of platelets in each field. Microscopy confirmation verified that the purified platelet had a leukocyte contamination < 0.02 % and a red blood cell contamination of < 1 %.
