# Supplementary figures and images for: Combining patient proteomics and in vitro cardiomyocyte phenotype testing to identify potential mediators of heart failure with preserved ejection fraction
Source: J Transl Med. 2016 Jan 20;14:18. doi: 10.1186/s12967-016-0774-3 (PMC4719542; doi:10.1186/s12967-016-0774-3)

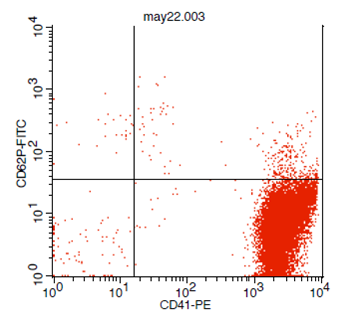

Supplement: Supplementary file 1 — 10.1186/s12967-016-0774-3 Flow cytometry to assess platelet activation. Purified platelet samples were incubated with fluorescently labeled antibodies against CD62P and CD41 and subject to flow cytometry. Platelets positive for CD41 + but negative for CD62P are non-activated. CD62P positive platelets are activated. [file 12967_2016_774_MOESM1_ESM.tif]

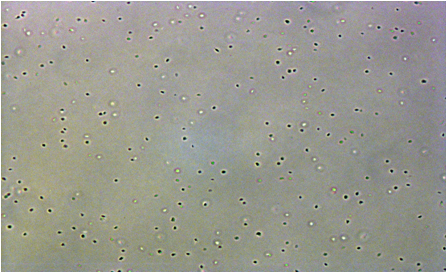

Supplement: Supplementary file 2 — 10.1186/s12967-016-0774-3 Microscopy for Platelet Purity. Isolated platelets were observed under the microscope. Visible red blood cells and leukocytes were counted and calculated as a percentage of platelets in each field. Microscopy confirmation verified that the purified platelet had a leukocyte contamination < 0.02 % and a red blood cell contamination of < 1 %. [file 12967_2016_774_MOESM2_ESM.tif]
